# Analysis of key genes and pathways of knee osteoarthritis based on GEO data mining

**DOI:** 10.1097/MD.0000000000046250

**Published:** 2025-11-28

**Authors:** Yuan Gao

**Affiliations:** a People’s Hospital Affiliated to Fujian University of Traditional Chinese Medicine, Fuzhou, China.

**Keywords:** GEO data mining, key biological process, key genes, key pathways, knee osteoarthritis

## Abstract

This study aims to give significant information for the research and treatment of knee osteoarthritis (KOA) targets, using bioinformatics methods to uncover the associated genes of KOA and explore their expression and clinical importance. GEO database was used to analyze differential genes of KOA, R was used to perform pathway enrichment analysis and functional annotation of differential genes, a protein–protein interaction network of differential genes was constructed, core modules of the protein–protein interaction network of relationship targets were screened, and common genes of 2 protein relationships were obtained. After combining, correcting, and screening the GSE51588, GSE55457, and GSE82107 data sets, 488 differential genes and 297 disease intersection targets were identified. The gene ontology study primarily focused on neutrophil activation and neutrophil activation in immunological response. Depending on Kyoto encyclopedia of genes and genomes analysis, signal pathways mostly included MAPK, IL-17, Wnt, TNF, and other information pathways. Biomarkers and treatment targets of KOA are likely to be PPBP, FPR2, PF4, ELANE, ORM1FPR1, PENK, POMC, RETN, ARG1, HIST1H2BB, and HIST1H2BJ. KOA can be treated clinically by regulating the expression of the above targets, activating or inhibiting related signal pathways, anti-inflammation, regulating apoptosis, and lowering oxidative stress.

## 1. Introduction

Knee osteoarthritis (KOA) is a chronic joint disease that is characterized by degeneration of the cartilage of the knee joint, subchondral bone remodeling and synovial inflammation leading to reduced mobility, pain, and stiffness throughout the joint.^[1]^ The disease is most common in middle-aged and elderly people, and the aging population has led to an increasing incidence of the disease.^[2]^ Currently, Western medical treatments are mainly based on nonsteroidal anti-inflammatory drugs and joint replacement.^[3]^ Although the above treatments can reduce the symptoms, there are adverse events such as gastrointestinal reactions, cardiovascular.^[4]^ In recent years, the unique therapeutic advantages of Chinese medicine have gradually been realized and valued.^[5,6]^ Although the current treatment for KOA is more extensive, there are still more or less shortcomings in terms of effectiveness.^[7]^ Modern medicine believes that the pathogenesis of KOA may be related to genetic, biological and biomechanical factors, and is also a major nuisance affecting the quality of human life.^[7]^ Although there are several treatment options for KOA, effective clinical management of KOA has been limited by suboptimal preclinical models and lack of accurate early diagnostic biomarkers. Therefore, the study of biomarkers associated with bone and joint pathogenesis and progression, further molecular identification is particularly important for both basic and clinical studies of KOA, and the discovery of new and effective prognostic biomarkers of KOA in order to avoid the early onset and progression of KOA. In addition, despite the existence of studies on KOA in the GEO database, they all suffer from the drawbacks of small samples that are not deeply mined and analyzed to draw conclusions. In this study, we proposed to extract multiple KOA gene chip datasets from the Gene Expression Database (GEO database) for differential gene expression profiling, to identify differential genes associated with osteoarthritis of the knee, and to functionally annotate the screened differential genes, so as to provide some reference for the study of the pathogenesis and therapeutic targets of KOA.

## 2. Materials and methods

### 2.1. Knee osteoarthritis differentially expressed genes (DEGs) screening, dataset integration, and batch normalize correction

The keyword “Knee osteoarthritis” was used to screen the relevant datasets in the GEO DataSets database.^[7]^ The obtained datasets were merged using Perl, and the batchNormalize correction was performed on the merged datasets using the sva and limma packages of R software. Then the Bioconductor software package was used for data processing and statistical analysis, and the expression data at the probe level were converted to gene symbol level data using the Perl language, and log2FC > 1 or log2FC < ‐1 and *P* < .05 were used as the screening conditions for candidate differential genes, and the limma package of the R software was used to analyze KOA tissues and normal human tissues for the microarray data for differential genes and performed DEG screening. The top 40 differential genes with the most obvious expression were also visualized.

### 2.2. Construction of protein–protein interaction (PPI) network and screening of key targets in KOA

A common target PPI network of DEG-related targets in KOA was mapped using the BisoGenet plug-in in Cytoscape 3.7.2. In order to explore the functional association of DEG, the 40 intersected genes obtained as described above were analyzed by the STRING database with the criteria of “Homosapiens” and the highest confidence level (0.70).^[8]^ The PPI network was obtained from the STRING database (https://string-db.org/), and the network relationships were subjected to PPIstat to obtain the protein-interaction network relationship map. CytoNCA plug-in was used to further analyze the core targets in the network and screen the nodes with degree and betweenness exceeding the median in the PPI network, so that the nodes could be used as the “key targets” for the subsequent research in terms of the amount of information transferred by the nodes and the efficiency of the transfer.

### 2.3. Gene ontology (GO) analysis and Kyoto encyclopedia of genes and genomes (KEGG) pathway enrichment analysis

GO enrichment analysis and KEGG pathway enrichment analysis of common targets of DEG were performed by loading clusterProfiler, enrichplo and org.Hs.e.g..db packages through R software, setting *P* < .05 as the filtering condition and q = 0.05 as the corrected filtering condition, and plotting the relevant graphs to take the most relevant pathways for mapping. According to the analysis of enrichment factor values to explore the biological function of DEG, to explore the possible biological function and signaling pathway mechanism of KOA.

## 3. Results

### 3.1. Screening of KOA differential genes

**Figure 1.**
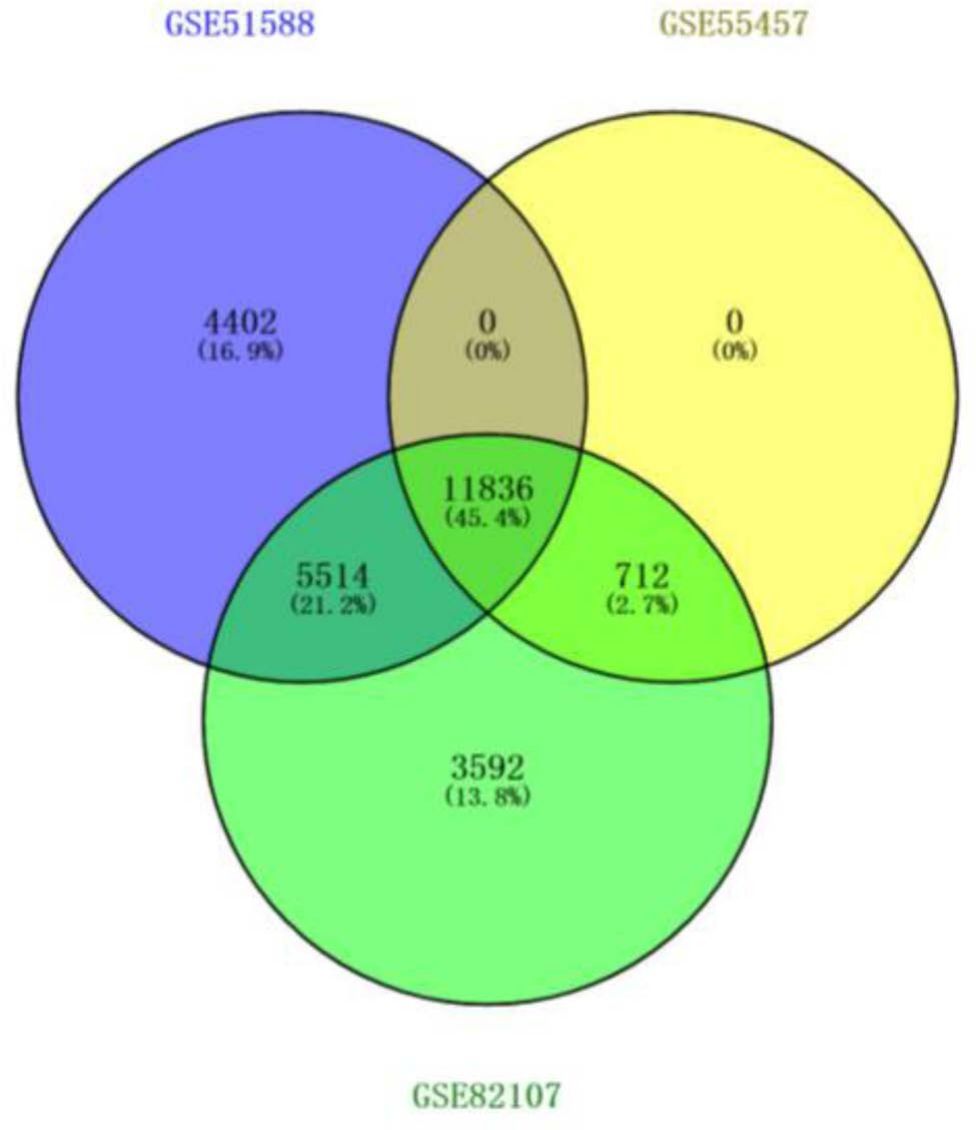
Wayne diagram of knee osteoarthritis disease targets for the GSE51588, GSE55457, and GSE82107 datasets.

**Figure 2.**
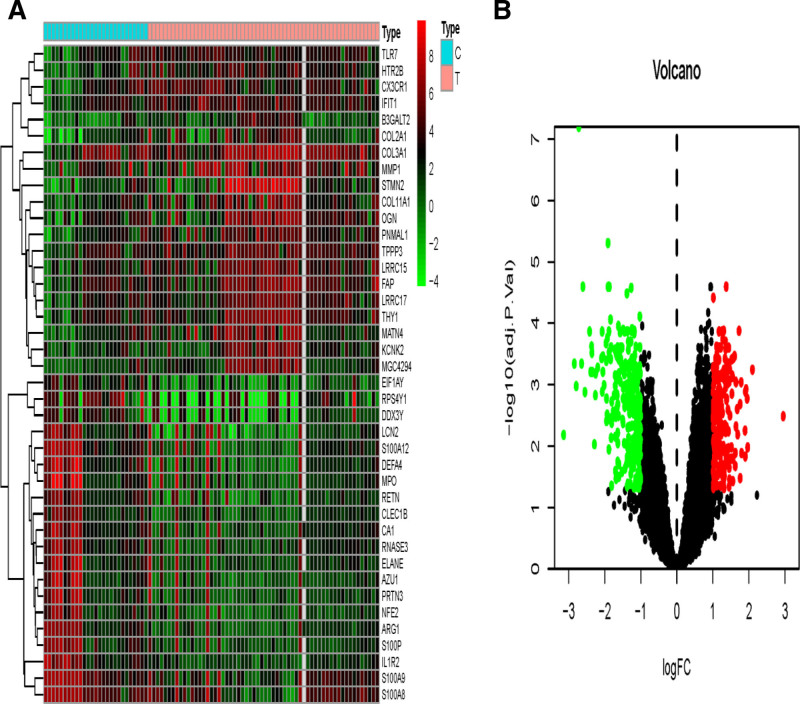
Differential expression of genes between osteoarthritic knee and normal tissues.

GSE51588, GSE55457, and GSE82107 datasets were obtained from the GEO microarray database, which contained 40, 10, and 10 primary KOA samples and 10, 10, and 7 normal human tissue samples, respectively. A total of 11,836 DEGs were identified from the 3 datasets by the R “sva” package with batchNormalize correction, and the data were combined in Figure 1. The 40 genes most closely associated with osteoarthritis of the knee were selected, and 488 DEGs were obtained, of which 215 (45.05%) were up-regulated and 273 (54.95%) were down-regulated, and the DEGs are shown in Figure 2, which lists a total of 40 genes that are likely to be closely associated with the pathogenesis of osteoarthritis of the knee.

### 3.2. PPI network construction and key target screening

**Figure 3.**
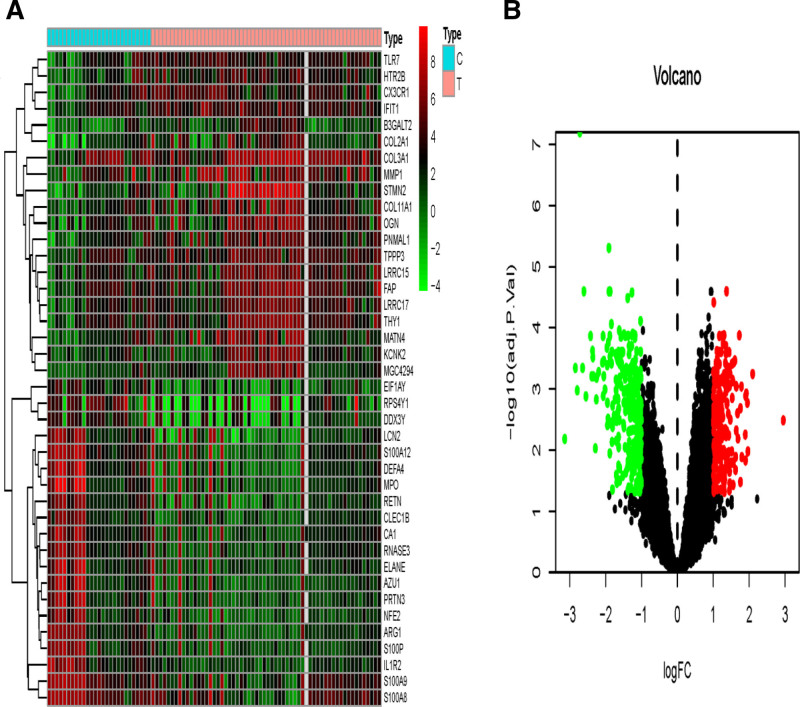
Protein interactions network relationships in osteoarthritis of the knee.

**Figure 4.**
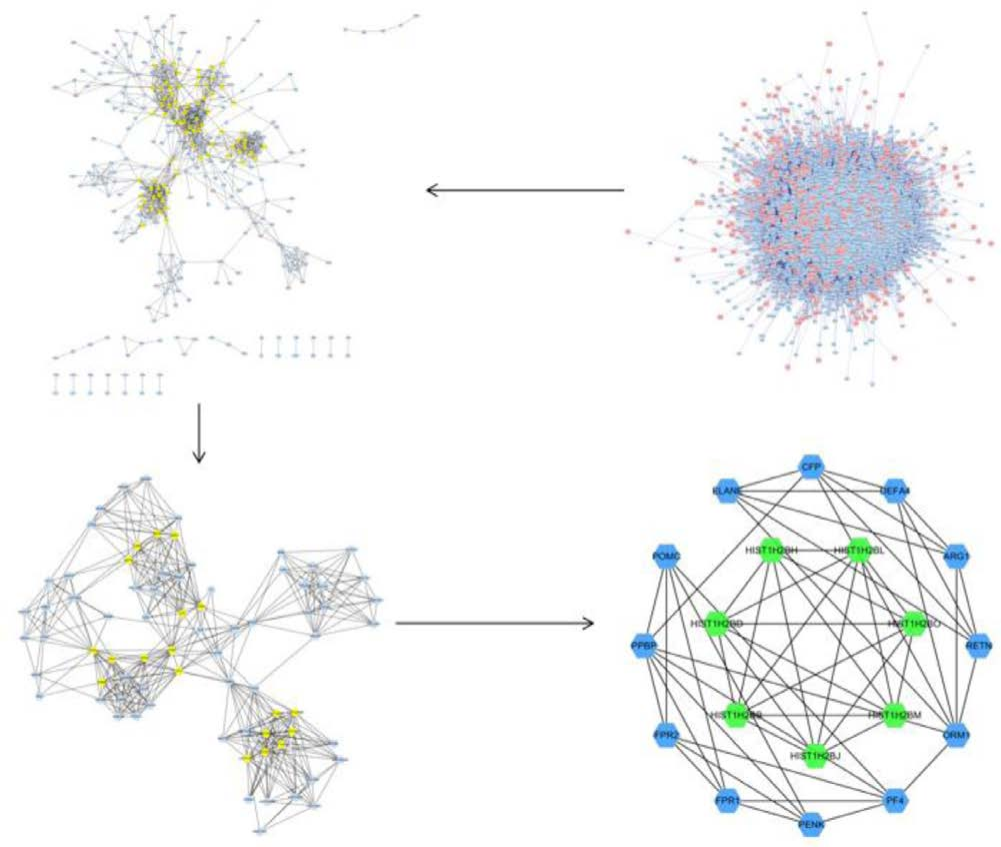
Map of key gene screening strategies for osteoarthritis of the knee.

Cytoscape 3.7.2 software was used to obtain 5336 directly or indirectly related targets and 123,346 inter-target interrelationships of the target PPI network diagram, and the network relationships were subjected to PPIstat to obtain the protein interactions network relationship diagram. It was found that the number of connections >20 were PPBP, FPR2, PF4, ELANE, CXCR4, ORM1, THBS1, FPR1, PENK, POMC, RETNARG1, COL3A1, HIST1H2BB, and HIST1H2BJ, see Figure 3. The intersection target interactions computed from the String database were then visualized, the intersection of the above PPI network graphs were extracted with CytoNCA toolkit and screened twice to find RM1, PPBP, HIST1H2BO, CFP, HIST1H2BL, PF4, ARG1, RETN, FPR2, etc, as the main initiating genes, see Table 1 and Figure 4. PPBP, FPR2, PF4, and ELANE were found, ORM1FPR1, PENK, POMC, RETN, ARG1, HIST1H2BB, and HIST1H2BJ were found to be the common genes, which shows that the above genes may be the potential targets for the pathogenesis of KOA.

**Table 1:** Topological parameters of the core target of the PPI network for osteoarthritis of the knee.

Gene name	Target site name	Connectivity	Lie between	Tightness
ORM1	Alpha-1-acid glycoprotein 1	7	34.33333333	0.121621622
PPBP	Platelet basic protein	7	34.33333333	0.121621622
HIST1H2BO	Histone H2B type 1-O	6	0	0.076923077
HIST1H2BB	Histone H2B type 1-B	6	0	0.076923077
HIST1H2BM	Histone H2B type 1-M	6	0	0.076923077
HIST1H2BH	Histone H2B type 1-H	6	0	0.076923077
CFP	Properdin	6	14.66666667	0.120805369
HIST1H2BL	Histone H2B type 1-L	6	0	0.076923077
HIST1H2BD	Histone H2B type 1-D	6	0	0.076923077
HIST1H2BJ	Histone H2B type 1-J	6	0	0.076923077
PF4	Platelet factor 4	6	14.66666667	0.120805369
ARG1	Arginase-1	5	0	0.116883117
RETN	Resistin	5	0	0.116883117
FPR2	N-formyl peptide receptor 2	5	0	0.116883117
DEFA4	Neutrophil defensin 4	5	0	0.116883117
FPR1	fMet-Leu-Phe receptor	5	0	0.116883117
POMC	Pro-opiomelanocortin	5	0	0.116883117
ELANE	Neutrophil elastase	5	0	0.116883117
PENK	Proenkephalin-A	5	0	0.116883117

PPI = protein–protein interaction.

### 3.3. Analysis of biological functions (GO)

**Figure 5.**
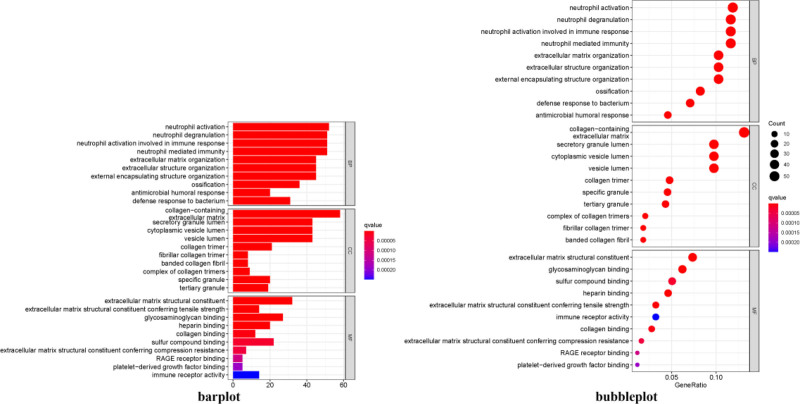
GO analysis of key genes in osteoarthritis of the knee. GO = gene ontology.

GO function analysis was performed by the R software package “clusterProfiler,” which classified the 27 co-targets into molecular function (MF), biological process (BP), cellular component (CC), as shown in Figure 5. The 27 common targets were analyzed by GO function according to the biological classification of MF, BP, and CC, and are shown in Figure 5, which shows that the BP changes of DEGs were mainly in the areas of neutrophil activation, neutrophil activation involved in immune response, neutrophil degranulation polymerase II promoter, etc. MF changes were mainly concentrated in the extracellular matrix structural constituent, glycosaminoglycans, and the MF changes were mainly concentrated in the extracellular matrix structural constituent, glycosaminoglycans, and the MF changes were mainly concentrated in the extracellular matrix structural constituent, glycosaminoglycans, and the MF changes. MF changes were mainly concentrated in extracellular matrix structural constituent, glycosaminoglycan binding, etc. CC changes were mainly enriched in collagen-containing extracellular matrix, secretory granule lumen, secretory granule lumen, and secretory granule lumen. CC changes were mainly enriched in collagen-containing extracellular matrix, secretory granule lumen, cytoplasmic vesicle lumen, and so on.

### 3.4. Screening of core pathways in KOA

**Figure 6.**
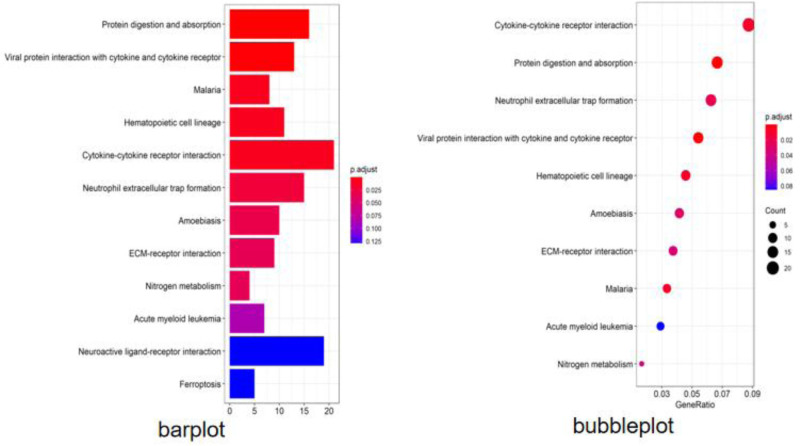
KEGG analysis of key genes in osteoarthritis of the knee. KEGG = Kyoto encyclopedia of genes and genomes.

**Figure 7.**
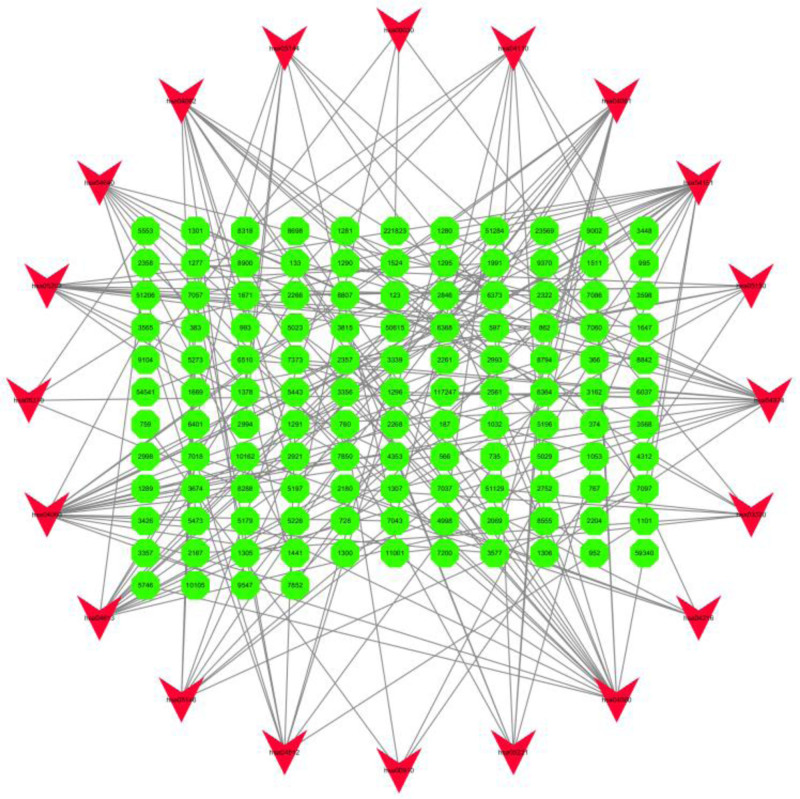
KEGG–target gene interaction network in osteoarthritis of the knee. KEGG = Kyoto encyclopedia of genes and genomes.

KEGG enrichment analysis using R language software yielded the following pathways, see Figure 6, mainly including protein digestion and absorption, MAPK signaling pathway, calcium signaling pathway, cytokine–cytokine receptor interaction, and other information pathways. The interactions between KEGG and target genes were visualized using Cytoscape 3.7.2 software, as shown in Figure 7, suggesting that the pathogenesis of osteoarthritis of the knee is achieved by acting on multiple signaling pathways, targets, and pathways.

## 4. Discussion

Osteoarthritis of the knee is more common in people over 50 years of age and is more common in women than in men, and the prevalence of the disease has increased significantly in China due to the increase in life expectancy and body mass index caused by the aging of the population.^[9]^ The main clinical manifestations of the disease are limb mobility, difficulty in movement and pain and swelling around the joints, which are more obvious after waking up in the morning.^[10]^ Current clinical treatments are mainly symptomatic, and common nonsteroidal anti-inflammatory and analgesic drugs are fast-acting but prone to recurrence.^[11]^ As for surgery, it is difficult, expensive and has a slow prognosis.^[12]^ Therefore, exploring biomarkers and therapeutic targets for KOA will be more urgent. The aim of this study was to use bioinformatics to comprehensively and integratively search for a clearer etiology and molecular mechanism in the pathogenesis of osteoarthritis of the knee independently of the traditional theory of cartilage pathogenesis, with a view to providing guidance for the subsequent search for molecular therapeutic targets for osteoarthritis of the knee.

In this study, a total of 488 DEGs of KOA were analyzed using bioinformatics techniques, and the central DEGs including PPBP, FPR2, PF4, ELANE, ORM1FPR1, PENK, POMC, RETN, ARG1, HIST1H2BB, and HIST1H2BJ were screened. Functional annotation of the biological processes of these 11 key genes showed that PENK and ORM1FPR1 were found to be up-regulated genes, and the other 9 genes were found to be down-regulated genes. Previous studies have shown that the above genes are involved in the disease progression of KOA.^[13]^ KOA, the most common aseptic inflammatory disease, secretes a large number of inflammatory transmitters and cytokines when the body undergoes a strong and abnormal inflammatory response, causing abnormal changes in anabolism and catabolism in the body.^[14]^ PPBP and PF4 are chemokines, FPR2 is a cytokine, FPR2 is a cytokine, and FPR2 is a cytokine. chemokines and FPR2 is a chemokine receptor. Chemokines are considered to be a type of pro-inflammatory cytokine that can induce cells of the immune system to enter the site of infection during the immune response. PPBP (chemokine protein) is involved in the pathological process of the onset, development and prognosis of KOA with a variety of inflammatory transmitters and cytokines that are released in response to a strong and abnormal inflammatory response in the organism.^[15]^ PF4 (platelet factor 4) directs immune cells to chemotaxis to protect the organism and remove foreign substances such as pathogens when they invade the body.^[16]^ FPR2 (formyl peptide receptor 2) is involved in physiological and pathological processes such as defense responses, inflammation, tumors, diseases related to disorders of glycolipid metabolism, and is a potential target for the treatment of a variety of diseases such as osteoarthritis of the knee.^[17]^ This suggests that exploring the study of the mediating role of inflammatory transmitters and cytokines in the pathogenesis of osteoarthritis of the knee may be an effective potential direction.

GO enrichment analysis showed that the key targets of KOA are mainly involved in biological processes such as neutrophil activation, neutrophil activation involved in the immune response, neutrophil degranulation polymerase II promoter, structural components of extracellular matrix, structural components of extracellular matrix with tensile strength, glycosaminoglycan binding, and collagens in extracellular matrix. The main target of the pathological process of KOA is articular cartilage. As the disease progresses, the accumulation of MMP1 degrades type II collagen in the cartilage matrix, thereby increasing the damage to the extracellular matrix and accelerating cartilage destruction in the knee joint.^[18]^ At the same time, the damaged tissues release inflammatory factors to inhibit anabolic and stimulate catabolism, ultimately leading to cartilage loss due to proteoglycan depletion to exacerbate the development of KOA.^[19]^ This is consistent with the results we obtained for bioprocesses enriched in inflammatory factors, collagen of the extracellular matrix and proteoglycans.

KEGG enrichment analysis showed that the key targets were mainly MAPK signaling pathway, IL-17 signaling pathway, calcium signaling pathway, protein digestion and absorption signaling pathway, Wnt signaling pathway, cytokine receptor interaction signaling pathway, TNF signaling pathway, p53 signaling pathway, hepatocellular carcinoma signaling pathway, HIF-1 signaling pathway and other information pathways. The functions of signaling pathways are mainly as follows: (1) Participate in cell cycle. *Calcium signaling pathway*: When the cells are stimulated by physical and chemical stimuli, it can affect the calcium ion pathway so that the physical and chemical signals can be transformed into biological signals and then affect the chondrocyte differentiation.^[20]^ In addition, it has been found that calcium ions can influence nutrient supply, joint osmotic pressure and chondrocyte apoptosis through movement in the joints.^[21]^ *MAPK signaling pathway*: Inflammatory factors produced in KOA patients can activate the MAPK pathway to increase the level of matrix metalloproteinases such as MMP1 and destroy articular chondrocytes,^[22]^ and may also affect synovial cells by regulating intra- and extracellular ion metabolism. It may also affect the proliferation, differentiation, movement and apoptosis of synovial and chondrocyte cells by regulating intracellular and extracellular ion metabolism, gene transcription and other links.^[23]^ *Wnt signaling pathway*: GaoYan Kuang et al^[24]^ found that the maintenance of chondrocyte function and value-added differentiation are regulated by Wnt and other signaling pathways, and that activation or blocking of Wnt signaling pathway to regulate its expression can improve cartilage degeneration, and thus reduce the progression of KOA lesions. The activation or blockade of Wnt signaling pathway can improve cartilage degeneration and reduce the progression of KOA. *p53 signaling pathway*: p53 is a tumor suppressor protein that inhibits cell cycle progression and regulates the expression of various genes such as apoptosis.^[25]^ It has been found that p53 may influence the progression of inflammation by regulating apoptosis in chondrocytes.^[26]^ *HIF-1 signaling pathway*: hypoxia-inducible factor 1 is an important factor in angiogenesis and immune regulation and is involved in a wide range of physiological processes such as vascular neovascularization, cell cycle and energy metabolism to repair damage to chondrocytes and synovial cells under hypoxia.^[27]^ (2) Inflammation-related. *IL-17 signaling pathway*: IL-17 is a class of inflammatory factors closely related to autoimmune diseases and is closely related to inflammatory response and cytokine production after tissue injury.^[28]^ Li Jingkun^[29]^ found that JNK/c-JunI and p38/c-Fos pathways can activate the transcription factor AP-1 by L-17A and up-regulate the expression of COX-2 and the production of PGE2, thus playing an inflammatory effects in tissue injury. *TNF signaling pathway*: TNF pathway is involved in cell growth, proliferation, inflammation and immunity, etc. Activated TNF pathway can induce NF-KB to enter the cell nucleus accordingly, which activates the downstream NF-κB signaling pathway and further promotes the production and release of inflammatory factors, such as TNF-α, IL-8, IL-6, etc., and ultimately aggravates the inflammatory response of the body.^[30]^ *MAPK signaling pathway*: When the cells are stimulated by the outside world, it will lead to the phosphorylation of a series of kinases upstream of MAPK, which will eventually become phosphorylated P38MAPK, phosphorylated ERKMAPK, and phosphorylated JNKMAPK through 2-site phosphorylation and activate the MAPK signaling pathway to take part in the process of inflammation, thus up-regulating the expression of inflammatory cells such as TNF-α and IL-6.^[31]^ In addition, protein digestion and absorption, cytokine receptor interaction, hepatocellular carcinoma and other signaling pathways have been rarely mentioned in the study of KOA, which may be a new research direction.

In summary, in this study, we systematically analyzed the mechanism of KOA disease development through a bioinformatics approach combining gene expression profiling and microarray data, and screened 488 DEGs and 11 pivotal genes, which may be potential biomarkers for KOA. Inflammation, damage to extracellular matrix, apoptosis of cartilage and synovial cells, and oxidative stress were found to be possibly involved in the pathophysiological alterations of KOA. The revelation of biomarkers will provide valuable information for research and therapeutic targets of KOA. However, there are some limitations in this study, and further experimental studies on the molecular mechanisms in conjunction with large samples and relevant serum and tissue protein analyses are needed to determine whether the key genes screened can be potential targets for KOA in the future.

## Author contributions

**Conceptualization:** Yuan Gao.

**Data curation:** Yuan Gao.

**Supervision:** Yuan Gao.

**Validation:** Yuan Gao.

**Visualization:** Yuan Gao.

**Writing – original draft:** Yuan Gao.

**Writing – review & editing:** Yuan Gao.
